# Caring for the Caregivers: A Narrative Review of Physician Health Protection Across High-Income Countries and Lessons for Low- and Middle-Income Countries

**DOI:** 10.7759/cureus.116850

**Published:** 2026-09-24

**Authors:** Hafza Bushra Razaq, Osama Saeed, Maksalmina Reshtin, Muhammad Usman Arshad, Waleed Usman, Nabiha Kashif, Muhammad Fawad Ul Hassan, Amna Sohail, Arham Amir Khawaja, Sundas Irshad, Haseeb Mehmood Qadri

**Affiliations:** 1 Physiotherapy and Rehabilitation, Punjab Institute of Neurosciences, Lahore, PAK; 2 Medical School, Frontier Medical and Dental College, Abbottabad, PAK; 3 Neurological Surgery, Punjab Institute of Neurosciences, Lahore, PAK; 4 Medical School, Shalamar Medical and Dental College, Lahore, PAK; 5 Internal Medicine, Allama Iqbal Medical College, Lahore, PAK; 6 Medical School, Mayo Hospital, Lahore, PAK; 7 Neurological Surgery, National Hospital and Medical Center, Lahore, PAK; 8 Family Medicine, Indus Hospital and Health Network, Lahore, PAK; 9 General Surgery and Surgical Oncology, Shaikh Zayed Hospital, Lahore, PAK; 10 General Surgery, Lahore General Hospital, Lahore, PAK

**Keywords:** burnout, developing countries, health policy, health workforce, high-income countries, low to middle income countries, occupational health, patient safety, physician health

## Abstract

Healthcare physicians (HCPs) across the world experience substantial mental, physical, and occupational health challenges, yet they remain an often-neglected group within healthcare systems. High levels of burnout, depression, anxiety, sleep disturbance, professional dissatisfaction, and even suicide have been reported in diverse settings, directly affecting patient safety and overall health system performance. This narrative review aimed to synthesize existing evidence on physician mental, physical, and professional health, with particular attention to similarities and differences between high-income countries (HICs) and low- and middle-income countries (LMICs). Peer-reviewed scientific articles, policy documents, and credible international reports examining physician well-being and workforce protection were analyzed. Recurrent themes across regions include burnout, depression, suicide risk, unsafe working conditions, professional dissatisfaction, migration, and collective actions such as strikes. In line with the objective of this review, the evidence also highlights important differences in how these challenges manifest and are addressed in HICs compared with LMICs.

Although many of these problems are present globally, their severity is often greater in LMICs because of weaker occupational protections, limited access to mental health services, financial insecurity, and structural constraints within under-resourced health systems. The review therefore emphasizes that physician health should be viewed as a system-level and ethical responsibility rather than an individual failing. It further proposes practical and policy-oriented strategies that LMICs can adopt, such as confidential support pathways, regulatory reforms, and leadership accountability, to strengthen physician well-being without requiring major infrastructure expansion.

## Introduction and background

Physicians play a central role in healthcare systems, yet the demands of modern medical practice increasingly place their own health and well-being at risk. Due to the demanding nature of medical practice, many physicians experience significant occupational stress, which can contribute to burnout. In many healthcare settings, physicians are often expected to appear resilient and self-reliant, which can make it difficult for them to openly seek help when they experience health or psychological challenges. Many doctors experience psychological distress but hesitate to seek support because of stigma, fear of professional consequences, and a culture that equates vulnerability with weakness [1]. Professional expectations and workplace culture may discourage physicians from prioritizing their own health or seeking support when they experience distress. Moral injury and emotional strain also remain significant concerns, including within advanced and well-structured healthcare systems [2]. In some healthcare settings, persistent occupational distress may become normalized, while institutional support for affected physicians remains limited.

Burnout is a global psychological condition characterized by physical and emotional exhaustion, depersonalization, and a declining sense of accomplishment [3]. Multiple risk factors are associated with burnout, affecting physicians’ perceptions, habits, attitudes, and interactions with patients. Work-related factors include excessive workload, long and frequent duty hours, zero tolerance for errors, detailed documentation, and loss of autonomy [4]. Personal factors include sleep deprivation [5], personal relationships, job struggles, finances, organizational issues [6], job insecurity, poor working conditions, smoking, and substance abuse [7]. The interaction of occupational and personal stressors may increase the risk of psychological distress and, in some cases, suicidal ideation and suicide risk among physicians [8].

Even the most advanced and structured healthcare systems continue to face challenges in addressing physician burnout. Targeted interventions such as online group coaching have demonstrated potential to reduce burnout among female resident physicians [9]. A staff consultation involving healthcare workers in a large public hospital identified sufficient staffing, adequate time, and appropriate working conditions as important requirements for supporting staff health and well-being [10-12]. Emergency physicians in Pakistan have reported severe burnout associated with workload and weak institutional support [13]. In contrast, high-income countries (HICs) have increasingly explored structured approaches to physician well-being, including leadership coaching and 360-degree feedback to support professional development and physician well-being [12]. These differences reflect broader variations in healthcare system capacity. HICs generally have greater financial and institutional resources to establish structured physician well-being and occupational health programs, whereas low- and middle-income countries (LMICs) often face workforce shortages, resource constraints, and limited access to formal mental health and occupational support. While several HICs have begun implementing structured initiatives to address physician burnout, evidence suggests that similar support systems remain limited or less developed in many LMICs [4,13].

This review examines the factors contributing to physician burnout and highlights differences in how these challenges are experienced and addressed in HICs and LMICs. These differences are important because physician distress is influenced not only by individual and occupational factors but also by the resources, workforce capacity, institutional policies, and support systems available within healthcare settings. While substantial research from HICs has informed the development of guidelines and institutional support systems, evidence from LMIC settings remains limited. Consequently, many existing recommendations are based on healthcare systems with greater financial resources and institutional capacity, which may not be directly applicable to LMIC contexts. By identifying key drivers of physician distress, this review aims to highlight practical areas for both immediate and long-term improvement. Early recognition of at-risk physicians and context-appropriate support strategies may improve physician well-being, patient care, and long-term health system sustainability.

## Review

Methodology

We conducted a narrative review to synthesize the heterogeneous evidence concerning physician health protection across HICs and LMICs. We assessed variables related to physician health, including epidemiological indicators such as burnout and suicide, workforce dynamics such as migration and strikes, as well as occupational health policies and institutional protections affecting physicians. Quantitative meta-analysis and Preferred Reporting Items for Systematic Reviews and Meta-Analyses (PRISMA)-guided systematic review methods were not used. Instead, we used structured thematic synthesis for analysis. Given the heterogeneous study designs, populations, outcomes, and policy sources included in this narrative review, the evidence was synthesized thematically rather than through quantitative pooling or formal systematic-review methodology.

Data Synthesis

The included evidence was synthesized descriptively according to physician health outcomes, occupational and organizational factors, workforce-related challenges, and institutional- or policy-level responses. Findings from HICs and LMICs were considered comparatively to identify common themes, contextual differences, and relevant health-system implications. Due to the heterogeneity of study designs, populations, outcomes, and evidence sources, quantitative pooling was not performed.

Search Strategy

The search strategy included a combination of subject terms and free-text terms combined using the Boolean operators “OR” and “AND.” The keywords used were as follows: physician health, burnout, suicide, LMICs, migration, and strikes (Table 1). The eligibility criteria are summarized in Table 2.

**Table 1:** Search strategy. LMICs: Low- and middle-income countries.

Database(s)	Search period	Search terms/keywords	Boolean operators used
Electronic databases (PubMed/MEDLINE, Scopus, and Google Scholar)	Database inception to March 2026	“Physician health,” “burnout,” “suicide,” “LMICs,” “migration,” “strikes”	Keywords were combined using OR within concepts and AND between concepts to identify relevant studies.

**Table 2:** Inclusion and exclusion criteria.

Inclusion criteria	Exclusion criteria
Studies focusing primarily on physicians and surgeons.	Studies focusing exclusively on non-physician healthcare professionals (e.g., nurses or allied health professionals), unless physicians were included in a comparative framework.
Studies conducted in high-income countries and/or low- and middle-income countries (LMICs).	Studies evaluating healthcare system performance without reporting physician-specific outcomes.
Studies examining physician health outcomes, including physical, mental, occupational, or psychosocial health.	-
Articles published in English or with an available English translation.	-

Review of evidence

The following section presents the findings identified from the reviewed literature, while interpretation and broader implications are addressed separately in the Discussion section.

Evidence From LMICs

The evidence from LMIC settings shows a consistent and concerning pattern. Across Iran, India, Uganda, Pakistan, and Lebanon, high levels of burnout, emotional exhaustion, and psychological distress are repeatedly reported. In some studies, nearly half or more of healthcare professionals demonstrated significant burnout symptoms, often linked to long working hours, staffing shortages, low autonomy, financial instability, and weak institutional support. Migration pressures were also strongly connected to dissatisfaction with pay and limited career growth, especially in South Asian and African contexts. Together, these findings suggest that distress in LMICs is not just an individual issue but a systemic one, driven by structural weaknesses and resulting in workforce attrition, reduced morale, and potential risks to patient safety (Table 3).

**Table 3:** Healthcare professional distress and workforce responses in LMICs. LMIC: Low- and middle-income countries; PPE: Personal protective equipment; OECD: Organisation for Economic Co-operation and Development.

Source of evidence	Study design	Country/region	Type of healthcare professional issue documented	Contributing factors	Key findings/specific results	Implications for the health system
Sharifi M et al. (2021) [14]	Systematic review	Iran	Burnout, anxiety, sleep deprivation	Long shifts, PPE shortage, pandemic workload	Marked increase in anxiety and sleep disturbance reported across multiple hospitals during the COVID-19 period.	Exposes the fragility of occupational protection systems and increases the risk of clinical errors during crises.
Mitra LG et al. (2024) [15]	Editorial/commentary	India	Work-life imbalance, psychosocial stress	Prolonged and stressful working hours, night and weekend duties, human-resource limitations	Highlights work-life imbalance, prolonged working hours, and workforce limitations as important contributors to healthcare worker stress and well-being.	Poor work-life balance may affect staff well-being and the continuity and quality of patient care.
Atukunda P et al. (2024) [16]	Observational study	Uganda	Work-life imbalance, fatigue	Staff shortages, excessive workload	Over one-third of participants reported severe fatigue perception linked to understaffing.	Reduces operational efficiency and increases the probability of preventable medical mistakes.
Ahmad W et al. (2018) [3]	Cross-sectional study	Pakistan	Burnout, professional dissatisfaction	Long duty hours, low autonomy	Approximately half of postgraduate trainees demonstrated high burnout scores.	Results in early career attrition and weakened clinical decision-making capacity.
Sarwar S et al. (2023) [13]	Cross-sectional study	Pakistan	Severe burnout, emotional stress	High workload, weak institutional support	More than half of emergency physicians perceived critical burnout levels.	Creates a risk of service disruption and compromises the quality of emergency care.
Youssef D et al. (2022) [8]	Survey study	Lebanon	Depression, suicide ideation	Delayed salaries, economic instability	A significant proportion of respondents expressed depressive symptoms during the financial crisis.	Threatens the continuity of national healthcare delivery and accelerates skilled migration.
Lohmann J et al. (2019) [4]	Systematic review	Multiple African countries	Psychological distress, burnout	Weak policy support, excessive workload	Burnout prevalence frequently exceeded 40% across reviewed LMIC settings.	Indicates chronic workforce instability and highlights the urgent need for systemic mental health safeguards.
OECD (2016) [17]	International policy report	South Asian countries	Workforce migration and retention challenges	Pay disparities, limited career growth, uneven working conditions	The report highlights persistent outward mobility of trained healthcare professionals from South Asian LMICs toward higher-income destinations and widening workforce gaps.	Results in long-term specialist shortages, loss of national training investment, and increased pressure on already under-resourced healthcare systems.

Evidence From HICs

In contrast, the evidence from HICs highlights structured responses rather than unchecked decline. Studies from the United States, the United Kingdom, and parts of Europe demonstrate that interventions such as peer coaching programs, confidential support systems, flexible scheduling policies, and national well-being frameworks can meaningfully reduce burnout, improve professional fulfillment, and strengthen retention intentions. Several randomized trials reported measurable improvements in fulfillment scores, reductions in impostor syndrome, and better psychological resilience following targeted programs. Although challenges remain in HICs, these findings show that organized policy action and leadership accountability can translate into tangible improvements for healthcare professionals and broader system stability (Table 4).

**Table 4:** Healthcare professional well-being policies and protective mechanisms in high-income countries. HIC: High-income countries; RCT: Randomized controlled trial.

Source of evidence	Study design	Country/region	Intervention/program implemented	Program mechanism	Specific results	Health system implications
Kiser SB et al. (2024) [18]	RCT	USA	Peer coaching program	One-to-one structured coaching sessions focusing on resilience and goal setting	Professional fulfillment scores improved by approximately 20% compared with the control group.	Supports clinician retention and promotes an emotionally stable workforce.
Mann A et al. (2023) [19]	Multisite RCT	USA	Digital group coaching	Online peer-group coaching with guided reflection exercises	Significant reduction in moral injury scores and an increase in flourishing indices were observed.	Provides a scalable psychological support model adaptable at the national level.
Fainstad T et al. (2022) [9]	RCT	USA	Resident group coaching	Gender-sensitive coaching and confidence-building modules	Impostor syndrome perception was reduced by nearly one-third among female residents.	Strengthens professional identity and reduces the risk of early career dropout.
Han S et al. (2019) [20]	Cost-analysis study	USA	Organizational investment in physician well-being and burnout prevention	Estimated economic impact of burnout through physician turnover and reduced clinical work effort	Physician burnout was estimated to cost US$4.6 billion annually, with approximately US$7,600 per physician lost due to turnover and reduced working hours.	Demonstrates that investing in physician well-being and retention strategies is economically beneficial, reducing workforce attrition and improving health system sustainability.
Pathmanathan A et al. (2023) [11]	Qualitative study	UK	Work-life balance and retention factors	Work-life balance, organizational support, control over working lives, and alternative career opportunities	Former doctors identified poor work-life balance, lack of support, limited control over working lives, and other workplace factors among the reasons for leaving medicine.	Addressing work-life balance, organizational support, and working conditions may help improve physician retention.
Tolins ML et al. (2023) [21]	Program evaluation	USA	Confidential peer support model	Anonymous support channels and early psychological intervention systems	Increased help-seeking behaviour and reduced stigma perception were observed.	Builds a psychologically safe institutional culture and early intervention pathways.
Korkmaz A et al. (2024) [22]	RCT	Turkey	Mindfulness breathing intervention	Structured breathing and relaxation exercises integrated into the work routine	A significant decline in anxiety scores was recorded post-intervention.	Demonstrates a cost-effective mental resilience strategy for large institutions.
National Health Service (2021) [23]	National policy	UK	National well-being framework	Leadership accountability, confidential counselling, and monitoring systems	System-wide adoption was associated with cultural transparency and early distress identification.	Promotes long-term workforce sustainability and institutional accountability.

Discussion

Doctors’ mental health should be understood as a problem at the system level rather than solely at the individual level. Research shows that burnout, emotional exhaustion, and depression are widespread across many healthcare settings [24,25]. In Pakistan, nearly half of the advanced trainees and more than half of the emergency doctors had severe burnout [3,13]. Similar findings were reported in Lebanon, Uganda, and India. Financial problems, compassion fatigue, and lack of personnel contributed to persistent psychological stress [8,15,16]. In LMICs, these problems may be exacerbated because weak systems and the absence of mental health policies can allow distress to build without adequate attention [4,26]. Stigma is also an issue [26]. Physicians may fear that disclosing a mental illness could damage their careers or reputations or result in loss of their license to practice, making them less likely to seek help. In Africa and South Asia, burnout rates exceeding 40% have been reported among physicians, but recognition and support remain limited [4,17,26].

Suicide among doctors is one of the hidden effects of unattended work-related stress. Doctors in Lebanon reported feelings of depression and suicidal thoughts during the economic crisis due to delayed payments and severe economic instability [8]. Emergency doctors in Pakistan with high levels of burnout and emotional distress are at risk of poor mental health [13]. In India, prolonged working hours, work-life imbalance, and psychosocial stress have been highlighted as important challenges affecting healthcare worker well-being [15]. However, physician suicide is still not discussed sufficiently in LMICs. Silence, institutional stigma, and the absence of standardized reporting systems may contribute to underrecognition of the problem. The connection between unattended depression, work-related stress, and suicidal ideation is evident, but healthcare systems may fail to use this information effectively to support their employees [8,13,24,26]. Troubled doctors may remain invisible because they lack private support or convenient access to care [26].

Work-related demands contribute to poor physical health among doctors, especially in lower-income countries. Long working hours, inadequate sleep, and poor-quality diets are common concerns among healthcare workers in Iran, Uganda, and Pakistan. These factors contribute to poor physical and mental health [3,14,16]. More than a third of healthcare workers in Uganda reported feeling extremely fatigued because of persistent staff shortages [16]. Iranian doctors experienced increased levels of anxiety and sleep disturbances during the COVID-19 era [14]. In many developing countries, employees find it difficult to access occupational healthcare or do not make sufficient use of it, leaving physical health issues inadequately addressed [17,27]. The COVID-19 pandemic worsened this situation, as doctors faced inadequate personal protective equipment, excessive workloads, and moral distress simultaneously [14].

Another factor contributing to physician distress is a maladaptive training culture associated with prolonged working hours. Compounding this concern is that such practices are often normalized and justified on the grounds that they are an integral component of postgraduate training necessary to become competent consultants [28]. This hostile culture may be transmitted from senior to junior physicians in a stepwise pattern, with junior doctors potentially repeating the same attitudes as they become senior, thereby perpetuating the cycle. This may significantly affect not only doctors’ attitudes toward patients, leading to compromised concentration and compassion [15], but also their decision-making capabilities and, in some cases, early career attrition [3,24,28].

These factors may contribute to physician attrition and workforce instability [27]. International migration may further compound workforce shortages in settings where physicians seek better working environments, career opportunities, remuneration, and living standards. The underlying drivers remain similar, resulting in the loss of national investment and trained healthcare personnel [17,27]. However, as immigration opportunities in major destination countries become more restricted, physicians may increasingly seek alternative pathways.

HICs have implemented several strategies to reduce physician burnout and improve the quality of care delivered. These include peer coaching programs, digital group coaching, resident group coaching, including studies conducted exclusively among female resident physicians, breathing interventions, recruitment of additional staff to reduce workload, and flexible scheduling policies. These strategies address multiple aspects of work-related challenges faced by healthcare workers [9,11,18,19,20,21]. While some interventions have proven highly effective, others suggest that a single strategy alone may be insufficient and that multiple combined interventions are often necessary to target different dimensions of well-being [9,29,30,31]. Separating treatment from disciplinary actions may encourage physicians to seek help for burnout without fear of punishment [28]. Studies have also shown that peer-support programs using stepwise frameworks can foster a more acceptable and psychologically safe institutional culture and provide earlier intervention pathways compared with traditional approaches such as employee assistance programs and psychological counselling [20,30,31]. Furthermore, poor work-life balance, lack of autonomy and altruism, and high workload have been identified as major contributors to physician burnout in HICs [11,25].

Although the reasons for excessive burnout among healthcare providers may overlap across high-, middle-, and low-income countries, long duty hours, high workload, and low salaries are key factors that healthcare organizations in middle- and low-income settings should prioritize when developing comprehensive work-life balance policies. Access to counselling, wellness programs, adequate staffing, and limited overtime may improve performance and well-being [29,30,31]. Promoting a supportive organizational culture, especially in middle- and low-income countries, is essential to encourage work-life balance and reduce negative judgment [16,26,29].

Physician health and patient safety are closely interconnected. Evidence consistently links physician exhaustion to reduced clinical judgment [3,24], an increased probability of medical errors [16,24], and consequently compromised professionalism. Increased workload due to understaffing and long duty hours may lead to emotional detachment, lack of empathy [15], and clinical inefficiency. In such low- or middle-income contexts, a distressed physician is not merely an individual concern but may become a systemic patient-safety hazard. These risks are amplified in resource-limited systems with minimal institutional support and financial instability. From an ethical point of view, health systems have a duty to protect caregivers, making the safeguarding of physician well-being both a moral obligation and a prerequisite for safe practice [27,28]. Drawing on protective mechanisms successfully incorporated in HICs, similar yet pragmatic reforms may be implemented by institutions to achieve comparable improvements [29-31].

Limitations

This review has several limitations that should be acknowledged. First, as a narrative synthesis, the study does not follow the strict methodological structure of a systematic review, which may limit the comprehensiveness of study selection. Second, much of the available literature on physician health originates from HICs, while evidence from LMICs remains comparatively limited. This imbalance may influence the depth of comparisons between regions. Third, the included literature is notably heterogeneous, particularly in terms of study designs and results. Despite these limitations, the review provides a broad perspective on physician health challenges and highlights important areas for future research and policy attention.

Recommendations

Low-cost yet high-impact interventions can be implemented in resource-constrained countries. Confidential peer-support groups should be established by hospitals to allow health professionals to express themselves in a safe space. Mental health referral pathways should be non-punitive and separated from disciplinary structures. Physician schedules should be streamlined to accommodate adequate rest periods during periods of intensive duty to reduce exhaustion. Institutions should offer leadership training in compassionate management to promote a healthy workplace environment.

At the policy level, confidentiality should be maintained regarding mental health disclosures during licensing to reduce stigma. There should be a clear differentiation between treatment and disciplinary processes to support professional growth. National physician well-being guidelines should be promptly adopted at every level to establish uniform standards across institutions.

Educational reforms are of equal significance. Medical education should be expanded to include mental health literacy in undergraduate and postgraduate curricula. Supervisors and senior physicians should be equipped to identify early signs of distress. Seeking medical help should be normalized and not framed as unprofessional. Finally, hospitals should invest in monitoring and data collection systems. Anonymous reporting, well-being surveys, and support activities should be incorporated into routine practices. Collaboration with medical associations can help identify potential risks and guide resource allocation (Figure 1).

**Figure 1:**
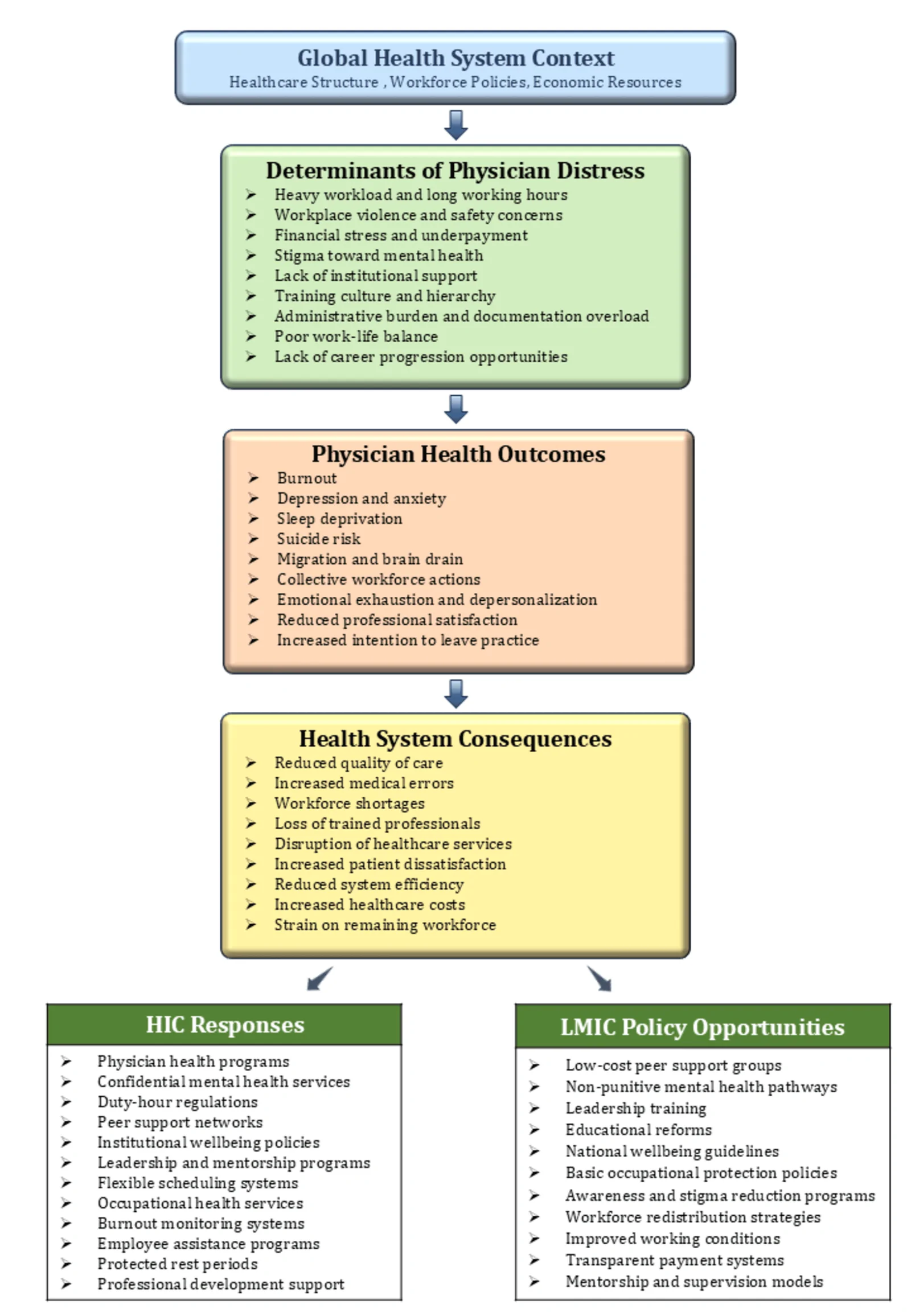
Author-proposed conceptual framework of physician health determinants and system-level consequences across high-income and low- and middle-income countries. Created using Microsoft PowerPoint, Version 16.x (Microsoft Corporation, Redmond, WA, USA) HICs: High-Income Countries; LMICs: Low- and middle-income countries.

## Conclusions

Existing evidence shows that there is a notably positive association between work-life balance and quality of care. Physicians comprise a central component of the healthcare system; lack of work-life balance and poor support systems put them at high risk of burnout. These risks are more pronounced in LMICs, where chronic resource constraints, workforce shortages, economic insecurity, and limited institutional resources make physicians more vulnerable to occupational challenges. Although HICs provide valuable resources, training, and support systems to alleviate these challenges, they are unable to offer perfect solutions. Physician health is vital for maintaining and delivering quality care, which can be achieved by implementing effective ethical and cultural measures rather than requiring large-scale infrastructure.

## References

[1] Romani M, Ashkar K (2014). Burnout among physicians. Libyan J Med.

[2] Clarke N, Crowe S, Humphries N, Conroy R, O'Hare S, Kavanagh P, Brugha R (2017). Factors influencing trainee doctor emigration in a high income country: a mixed methods study. Hum Resour Health.

[3] Ahmad W, Ashraf H, Talat A (2018). Association of burnout with doctor-patient relationship and common stressors among postgraduate trainees and house officers in Lahore-a cross-sectional study. PeerJ.

[4] Lohmann J, John D, Dzay A (2019). Prevalence and factors associated with poor mental health among healthcare professionals in low- and lower-middle-income countries: a systematic review protocol. Syst Rev.

[5] Carrieri D, Mattick K, Pearson M, Papoutsi C, Briscoe S, Wong G, Jackson M (2020). Optimising strategies to address mental ill-health in doctors and medical students: 'Care Under Pressure' realist review and implementation guidance. BMC Med.

[6] Menon NK, Shanafelt TD, Sinsky CA (2020). Association of physician burnout with suicidal ideation and medical errors. JAMA Netw Open.

[7] Harvey SB, Epstein RM, Glozier N (2021). Mental illness and suicide among physicians. Lancet.

[8] Youssef D, Youssef J, Abou-Abbas L, Kawtharani M, Hassan H (2022). Prevalence and correlates of burnout among physicians in a developing country facing multi-layered crises: a cross-sectional study. Sci Rep.

[9] Fainstad T, Mann A, Suresh K, Shah P, Dieujuste N, Thurmon K, Jones CD (2022). Effect of a novel online group-coaching program to reduce burnout in female resident physicians: a randomized clinical trial. JAMA Netw Open.

[10] Lawrence W, Hine J, Watson D, Smedley J, Walker-Bone K (2022). How to improve hospital employees' health and well-being: a staff consultation. BMC Health Serv Res.

[11] Pathmanathan A, Snelling I (2023). Exploring reasons behind UK doctors leaving the medical profession: a series of qualitative interviews with former UK doctors. BMJ Open.

[12] Schwartz R, Weimer-Elder B, Wilkins E (2022). Developing a feedback-rich culture in academic medicine: the effect of coaching and 360-feedback on physician leadership. BMC Med Educ.

[13] Sarwar S, Mahmood A, Raja MF, Mahmud Y (2023). Burnout and health related quality of life among emergency physicians working at emergency at tertiary care hospital in Lahore. Pak J Med Sci.

[14] Sharifi M, Asadi-Pooya AA, Mousavi-Roknabadi RS (2021). Burnout among healthcare providers of COVID-19; a systematic review of epidemiology and recommendations. Arch Acad Emerg Med.

[15] Mitra LG, Sharma J, Walia HS (2024). Improving work-life balance and satisfaction to improve patient care. Indian J Crit Care Med.

[16] Atukunda P, Nyamboga TO (2024). Striking a balance: investigating work-life balance strategies and their impact on healthcare provider performance in public health facilities, Bushenyi District, Uganda. Int J Res Innov Soc Sci.

[17] Organisation for Economic Co-operation and Development (2016). Health workforce policies in OECD countries: right jobs, right skills, right places. Paris: OECD Publishing.

[18] Kiser SB, Sterns JD, Lai PY, Horick NK, Palamara K (2024). Physician coaching by professionally trained peers for burnout and well-being: a randomized clinical trial. JAMA Netw Open.

[19] Mann A, Shah AN, Thibodeau PS (2023). Online well-being group coaching program for women physician trainees: a randomized clinical trial. JAMA Netw Open.

[20] Han S, Shanafelt TD, Sinsky CA (2019). Estimating the attributable cost of physician burnout in the United States. Ann Intern Med.

[21] Tolins ML, Rana JS, Lippert S, LeMaster C, Kimura YF, Sax DR (2023). Implementation and effectiveness of a physician-focused peer support program. PLoS One.

[22] Korkmaz A, Bernhardsen GP, Cirit B (2024). Sudarshan Kriya yoga breathing and a meditation program for burnout among physicians: a randomized clinical trial. JAMA Netw Open.

[23] (2021). National Health Service. NHS health and wellbeing implementation guide. https://www.england.nhs.uk/wp-content/uploads/2021/11/NHS-health-and-wellbeing-framework-implementation-guide.pdf.

[24] Tawfik DS, Profit J, Morgenthaler TI (2018). Physician burnout, well-being, and work unit safety grades in relationship to reported medical errors. Mayo Clin Proc.

[25] American Medical Association (2026). National physician burnout survey. American Medical Association.

[26] Tirandaz A, Nawaser K, Ramezani N (2025). Beyond the white coat: organizational strategies to address physician burnout and mental health. Iran J Public Health.

[27] O'Connell R, Hosain F, Colucci L, Nath B, Melnick ER (2024). Why do physicians depart their practice? A qualitative study of attrition in a multispecialty ambulatory practice network. J Am Board Fam Med.

[28] Accreditation Council for Graduate Medical Education (2025). ACGME Common Program Requirements (Residency). https://www.acgme.org/globalassets/pfassets/programrequirements/2025-reformatted-requirements/cprresidency_2025_reformatted.pdf.

[29] Kiratipaisarl W, Surawattanasakul V, Sirikul W (2024). Individual and organizational interventions to reduce burnout in resident physicians: a systematic review and meta-analysis. BMC Med Educ.

[30] Dyrbye LN, Shanafelt TD, Gill PR, Satele DV, West CP (2019). Effect of a professional coaching intervention on the well-being and distress of physicians: a pilot randomized clinical trial. JAMA Intern Med.

[31] West CP, Dyrbye LN, Rabatin JT (2014). Intervention to promote physician well-being, job satisfaction, and professionalism: a randomized clinical trial. JAMA Intern Med.

